# Ginseng Oligopeptides Improve the Intestinal Physiology and Promote the Antioxidant Capacity of the Gut-on-a-Chip Model

**DOI:** 10.3390/nu16060845

**Published:** 2024-03-15

**Authors:** Mei You, Meihong Xu

**Affiliations:** 1 Department of Nutrition and Food Hygiene, School of Public Health, Health Science Centre, Peking University, Beijing 100191, China; 2 Institute for Chronic and Non-Communicable Disease Control and Prevention, Ningxia Center for Disease Control and Prevention, Yinchuan 750004, China; 3 Beijing Key Laboratory of Toxicological Research and Risk Assessment for Food Safety, Peking University, Beijing 100191, China

**Keywords:** ginseng oligopeptides, ageing intestine, gut-on-a-chip

## Abstract

During ageing, the permeability of the intestinal barrier increases, the integrity of the intestinal barrier decreases, and the physiology of intestinal cells changes. Furthermore, intestinal inflammation and excessive oxidative stress are both likely to cause systemic diseases. Ginseng oligopeptides have a positive significant effect in terms of improving human health and delaying ageing, but their role in the ageing of the intestine has not been studied much. In our experiment, we constructed a gut-on-a-chip model and induced senescence of the chip with H_2_O_2_ so as to explore the effects of ginseng oligopeptides on the senescent intestine. The experimental results showed that ginseng oligopeptides had no obvious effects on the integrity of the intestine, including the TEER value and the expression of tight junction proteins. However, ginseng oligopeptides might have other positive effects, such as inhibiting excessive cell proliferation, promoting mucin secretion, and increasing the antioxidant capacity of the intestine, to improve intestinal health.

## 1. Introduction

In recent years, population ageing has become a trend in the developing world. Improving the health conditions of the elderly has become an important issue. The physiology of intestinal cells changes with age, and changes in intestinal secretion impair the normal physiological functions of the intestine [1]. Dysfunction of the intestinal barrier is considered to be a common pathophysiological feature of ageing organisms [2]. The epithelial tight junction is altered in ageing intestines [3], leading to an increase in intestinal permeability [4], and the balance of the intestinal flora is also disturbed [5,6]. The function of the intestinal barrier reduces with age, meaning that the elderly are less resistant to external pathogens, thus leading to increased levels of inflammation, and in severe cases, this may induce systemic inflammation [7]. The deceleration of intestinal function ageing also leads to a high incidence of digestive and neurological diseases and even systemic diseases in the elderly [8]. Therefore, maintaining intestinal health in the elderly is critical to improving the overall health of the body.

It is now believed that ageing is inextricably linked to oxidative stress, which is particularly important in age-related diseases [9]. The antioxidant capacity of ageing intestines decreases dramatically with age. It has been found that excessive oxidative stress in the intestine might induce apoptosis, necrosis, and cell cycle arrest, thus affecting the physiology of intestinal cells and leading to an incomplete intestinal barrier [10,11]. Meanwhile, excessive oxidative stress in the intestine triggers rapid transfer between gut microbiota, leading to alterations to the intestinal flora [12]. In addition, ageing is often accompanied by the development of chronic low-grade inflammation, which causes damage to organs and tissues.

Ginseng is a widely used functional food with applications in maintaining and improving human health. It has been found that ginseng can decrease levels of inflammation [13]. Ginseng polysaccharides also have obvious anti-inflammatory effects that might inhibit intestinal inflammation by regulating the intestinal flora and autophagy dysfunction [14]. In addition, other active components of ginseng also have health-promoting effects. Ginseng glycopeptides have some hypoglycemic effects [15], and ginseng proteins are able to inhibit cell apoptosis [16]. Ginseng oligopeptides (GOPs) are small-molecule bioactive peptides derived from ginseng which have a certain positive significance in promoting health. Our previous studies showed that GOPs have the effect of delaying cellular senescence [17,18], indicating that GOPs might have important anti-ageing effects. Previous studies have shown that GOPs are able to attenuate radiation-induced immune dysfunction and intestinal damage, possibly by reducing the levels of intestinal inflammation and oxidative stress [19,20]. In this experiment, we further investigated the effects of GOPs on intestinal health.

In this research, we tried to explore the influence of GOPs on the ageing intestine by constructing a gut-on-a-chip model. Organoids are widely used in modeling the development of diseases and the screening of drugs [21]. Currently, research on gut health is becoming a hot topic. Since the ethical limitations of traditional animal experiments and species differences hinder the results when generalizing them, gut organoids are becoming an efficient research method. It has been verified that the cellular compositions of gut organoids and the human intestine are similar [22]. Some studies have used gut organoids to simulate damaged or aged intestines, and they found that aged colonic organoids cultured from mouse tissues showed overall methylation changes similar to those of aged mouse colons [23,24]. All of the above suggests that organoids will become a powerful supportive tool for studying ageing intestines. In this experiment, we used a colon gut-on-a-chip to simulate the ageing of the intestine so as to find how GOPs intervene in the structure and function of the ageing intestine.

## 2. Materials and Methods

### 2.1. Subjects

In this experiment, we used the GOPs extracted from the Panax ginseng C.A. Meyer [25], and Jilin Taigu Biological Engineering Co., Ltd. (Jilin, China) supplied the GOP samples.

### 2.2. Culture and Treatments of the Gut-on-a-Chip

Frozen human colonic organoids were resuscitated, and expansion of the organoids was carried out. They were then inoculated on the chips for further culture. The colonic organoids were grown on the chips for about 5 days, and then the medium was changed to the differentiation medium to allow the intestinal cells to continue differentiation. Morphological and trans-epithelial electrical resistance (TEER) tests were performed during the growth and differentiation phases, and real-time fluorescence quantitative PCR (qPCR) was carried out to detect the differentiated cell types at the end of cell differentiation.

After the successful construction of the gut-on-a-chip, 50 μmol/L, 100 μmol/L, 200 μmol/L, 400 μmol/L and 800 μmol/L of H_2_O_2_ were added to the medium for 48 h. We evaluated the function of the chip after the H_2_O_2_ treatment. The TEER values, cell viability after the intervention and the gene content of cell markers p16 and p21 were all detected. Overall, the optimal H_2_O_2_ concentration of 200 μmol/L was selected by combining the results of the above tests.

In this study, three groups were established, including a normal control group, a H_2_O_2_ model group and a GOP intervention group. After the successful construction of the gut-on-a-chip model, a normal culture medium was used for 48 h without adding any interventions in the normal control group. In the H_2_O_2_ model group, after the construction of the model, a culture medium with 200 μmol/L H_2_O_2_ was used for 48 h. In the GOP intervention group, after the construction of the model, a culture medium with 200 μmol/L H_2_O_2_ and 400 μg/L GOPs was used for 48 h. We determined the dose of GOPs based on previous studies [17]. At the end of the experiment, all cells were collected for tests.

### 2.3. Observation of Morphological Structure

After 0, 4, 24 and 48 h of intervention, the morphology of the gut-on-a-chip was observed under a high-content confocal microscope (Molecular Device, Sunnyvale, CA, USA), and pictures were taken at the same time.

### 2.4. Detection of the Apparent Permeability Coefficient (Papp)

We used RPMI-1640 medium to prepare a working solution of 25 μg/ml fluorescent yellow. First, we replaced the middle and right holes of each unit of the chip with RPMI-1640 medium and the fluorescent yellow working solution, and incubated the chip for 3 h. Then, we used RPMI-1640 medium to dilute the working solution to obtain the standard curve solution for 8 concentration points. Next, we removed 100 μL per well for each concentration point for testing and set up 2 replicates for each concentration point. Also, 2 additional wells of RPMI-1640 medium were added for background detection. We used a microplate reader (BioTek, Winooski, VT, USA) to determine the value. The excitation wavelength was set to 428 nm and the emission wavelength was set to 536 nm. According to the fluorescence value of the standard curve solution and the configured concentration, we performed linear regression. The Papp value was calculated using the following formula:

(1) $$Papp=(\frac{1}{Permeability\times sample\;concentration})\times (\frac{supernatant\;concentration\times supernatantvolume}{Duration\;of\;intervention})$$

### 2.5. Detection of the TEER

The basal TEER of the chip was detected before the experiment and on days 2, 4, 5, 7, 9 and 10 of cell differentiation. We removed the chip before performing the assay; after which, we used a transmembrane resistance meter (Daxiang, Beijing, China) to detect the TEER of the chip. The TEER of the intestinal barrier was calculated using the following equation. The transmembrane area in this experiment was 0.07 cm^2^.

(2) $${TEER}_{Day\;N}=\left({TEER\;of\;the\;chip}_{Day\;N}-basal\;TEER\;of\;the\;chip\right)\times transmembrane\;area$$

### 2.6. Immunofluorescence Staining

We added 4% PFA solution to the lumen in the chip for cell fixation. After incubation, the primary and secondary antibody solutions were added to the chip; after which, DAPI was added to phalloidin solution (1:200) to perform staining. Fluorescence imaging of the chip was performed under DPBS immersion and images were captured using a high-content confocal microscope (Molecular Device, Sunnyvale, CA, USA).

### 2.7. QPCR Assay

The cell lysate was added to the chip and then was recovered from each well. RNA extraction was performed according to the instruction manual. The extracted RNA was transferred to PCR tubes, and the extracted total RNA was placed into cDNA libraries. Finally, the cDNA was subjected to qPCR of the target genes using a ChamQ Universal SYBR qPCR kit (Vazyme, Nanjing, China). Forward and reverse primers, cDNA and qPCR premix were added to each qPCR reaction, and three technical replicates were performed for each target gene. Table 1 is the list of PCR primer base sequences which were designed according to the open database of Prime bank.

### 2.8. Statistical Analysis

The results are shown as means ± standard deviation (SD). SPSS 24.0 software (SPSS, Inc., Chicago, IL, USA) was used for data analyses, and a one-way analysis of variance (ANOVA) was the main method we used. The least-significant difference (LSD) was used for the comparison within all groups, and *p* < 0.05 was the criterion for the significance of differences.

## 3. Results

### 3.1. Culture and Construction of the Ageing Gut-on-a-Chip Model

**Figure 1:**
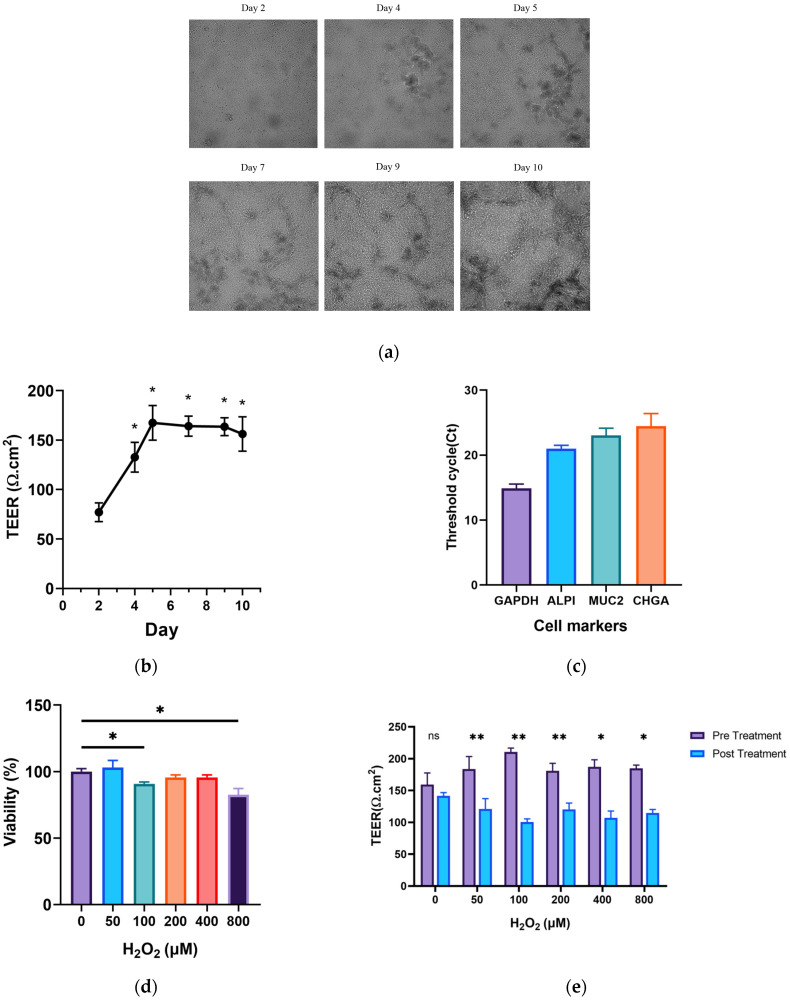

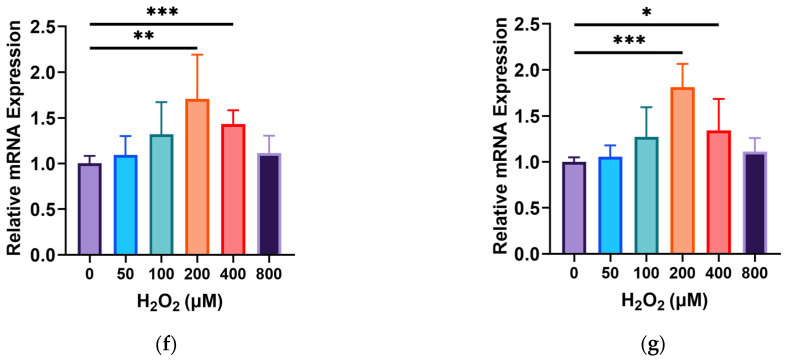
The barrier characteristics of the gut-on-a-chip model and the construction of the ageing gut-on-a-chip model. (**a**) The culture process of the chip (scale bar: 200 μm); (**b**) the TEER values during the culture of the chip; (**c**) differentiated cell markers of the chip; (**d**) cell viability of the chip; (**e**) changes in TEER values before and after H_2_O_2_ intervention; (**f**) expression level of p16; (**g**) expression level of p21. ^ns^ *p* > 0.05, * *p* < 0.05, ** *p* < 0.01, *** *p* < 0.001.

The chip was cultured according to the method described before. It took about 10 days for the chip to grow and differentiate into a complete barrier, as shown in Figure 1a. After starting the culture, the TEER of the chip barrier gradually increased. The chip barrier began to stabilize on the fifth day of the culture, and the TEER stabilized at the same time. On the 10th day of the culture, the chip showed strong expression levels of cell differentiated markers, such as CHGA, MUC2, ALP1, etc. This indicates that the chip mainly contained cup cells, absorptive epithelial cells and enteroendocrine cells. The intestinal absorptive epithelial cells were the most abundant cells on the chip. The barrier characteristics of the chip are displayed in Figure 1b,c.

As shown in Figure 1d, the cell viability of the 100 μmol/L and 800 μmol/L H_2_O_2_ intervention groups showed a significant decrease compared with the group without intervention (*p* < 0.05). The TEER of the chip barriers was evidently reduced in all H_2_O_2_ intervention groups (*p* < 0.05), as shown in Figure 1e. In addition, p16 and p21 are apparent markers of the cell cycle. We found that the gene expression of p16 and p21 of the chip was significantly up-regulated by the intervention of 200 μmol/L and 400 μmol/L H_2_O_2_ (*p* < 0.05); the effect of 200 μmol/L H_2_O_2_ was more obvious, as shown in Figure 1f,g.

### 3.2. Effects of GOPs on the Barrier Integrity of the Gut-on-a-Chip

**Figure 2:**
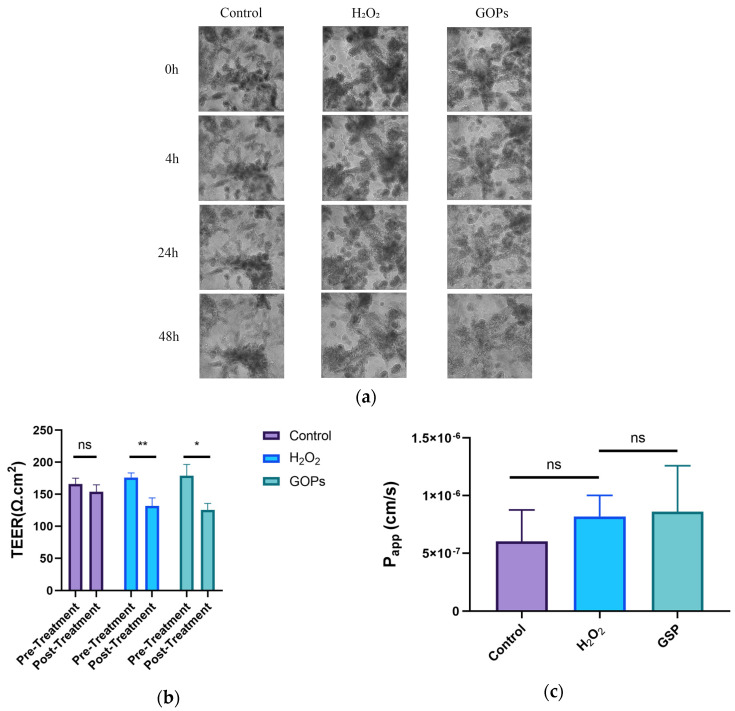
Variation in the integrity of the gut-on-a-chip. (**a**) The three-dimensional structure of the gut-on-a-chip model (scale bar: 200 μm); (**b**) changes in the TEER of the chip; (**c**) changes in the permeability of the chip. ^ns^ *p* > 0.05, * *p* < 0.05, ** *p* < 0.01.

The intestinal barriers of the chip in three groups remained intact, with no significant changes in the three-dimensional structures between the 48 h treatments, as shown in Figure 2a. As shown in Figure 2b, the TEER of the chip decreased significantly with H_2_O_2_ treatment (*p* < 0.05), but the GOP intervention group did not exhibit changes, in contrast with the H_2_O_2_ intervention group. The TEERs of the GOP intervention group and the H_2_O_2_ model group showed no statistical difference (*p* > 0.05). As shown in Figure 2c, the permeabilities of the chip in the three groups were not statistically different (*p* > 0.05).

### 3.3. Effects of GOPs on the Cell Physiology of the Gut-on-a-Chip

**Figure 3:**
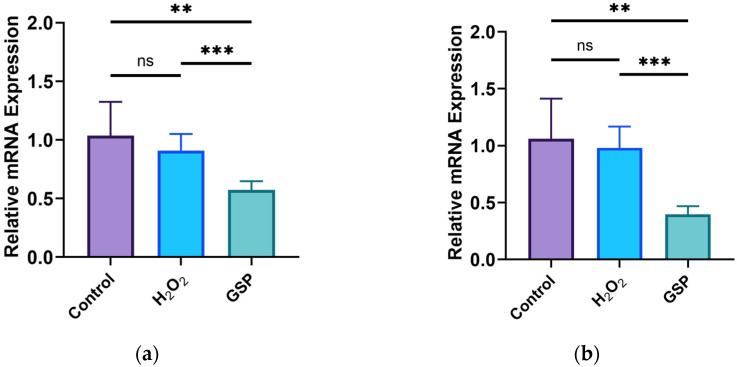

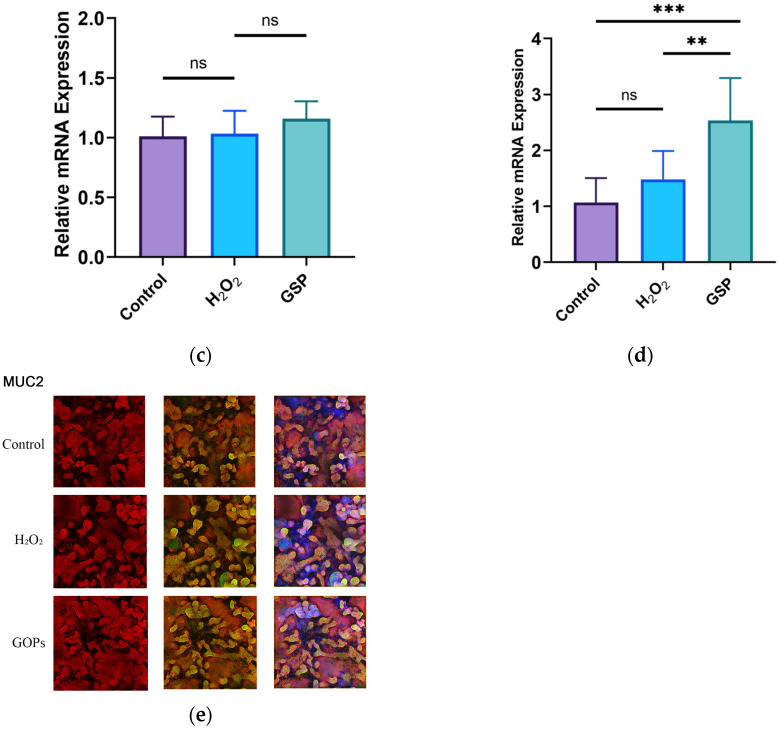
The cell physiology of the gut-on-a-chip. (**a**) Expression level of Ki67; (**b**) expression level of Lgr5; (**c**) expression level of glycoproteins; (**d**) expression level of MUC2; (**e**) immunofluorescence staining of cellular MUC2 (red: MUC2; green: cytoskeleton; blue: nucleus. Scale bar: 200 μm). ^ns^ *p* > 0.05, ** *p* < 0.01, *** *p* < 0.001.

The experimental results show that the expression of Ki67 and Lgr5 in the H_2_O_2_ model group did not change after the H_2_O_2_ intervention (*p* > 0.05), as shown in Figure 3a,b. In comparison with the H_2_O_2_ model group, the expression levels of both Ki67 and Lgr5 obviously decreased in the GOP intervention group (*p* <0.05). As shown in Figure 3c, the glycoprotein expressions were not statistically different between the three groups (*p* > 0.05). The expression of MUC2 had no statistical significance in the H_2_O_2_ model group and the normal control group (*p* > 0.05), but the MUC2 expression in the GOP intervention group exhibited a significant increase compared to the H_2_O_2_ model group and the normal control group (*p* < 0.05), as shown in Figure 3d. A cellular immunofluorescence analysis showed that cellular MUC2 was strongly expressed in the three groups, and it was localized on the surface of the cell membrane, as shown in Figure 3e.

### 3.4. Effects of GOPs on Tight Junctions of the Gut-on-a-Chip

**Figure 4:**
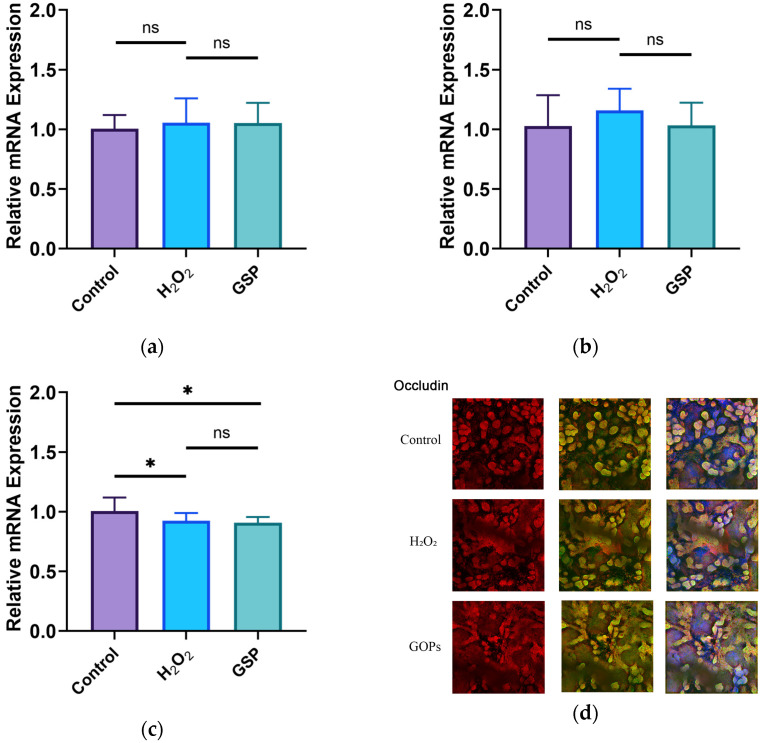
Expression of tight junction proteins. (**a**) Expression level of Occludin; (**b**) expression level of ZO-1; (**c**) expression level of Claudin-1; (**d**) immunofluorescence staining of cellular Occludin (red: MUC2; green: cytoskeleton; blue: nucleus. Scale bar: 200 μm). ^ns^ *p* > 0.05, * *p* < 0.05.

The results showed that the expression levels of Occludin and ZO-1 were not statistically different in the H_2_O_2_ model group and the normal control group (*p* > 0.05), but the expression level of Claudin-1 exhibited a decrease (*p* < 0.05), as shown in Figure 4a–c. We found that there was no significant change in the expression levels of tight junction proteins in the GOP intervention group compared with the H_2_O_2_ model group (*p* > 0.05). A cellular immunofluorescence analysis revealed that cellular Occludin was strongly expressed in all groups, and it was localized inside the cell, as shown in Figure 4d.

### 3.5. Effects of GOPs on Oxidative Stress and Inflammation of the Gut-on-a-Chip

**Figure 5:**
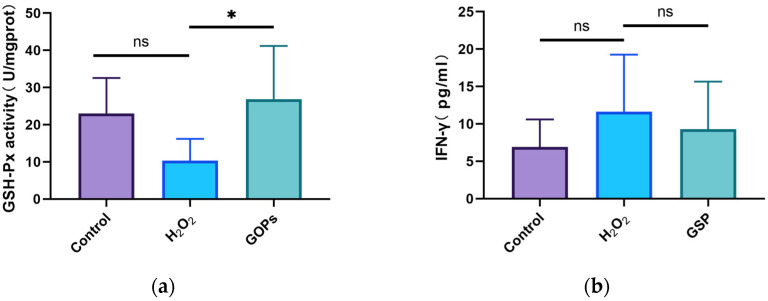
Expression of cellular oxidative stress and inflammatory markers. (**a**) Expression level of GSH-Px; (**b**) expression level of IFN-γ. ^ns^ *p* > 0.05, * *p* < 0.05.

As shown in Figure 5a, the activity of GSH-Px in the GOP intervention group was significantly higher compared to the H_2_O_2_ model group (*p* < 0.05). As shown in Figure 5b, the IFN-γ concentrations of the H_2_O_2_ model group, the normal control group and the GOP intervention group appeared to have no significant differences (*p* > 0.05).

## 4. Discussion

Currently, a number of researchers study the physiology or pathology of different organs via the organoids, which is a mature technique. Studies have shown that the genome of organoids is still stable and organoids maintain disease attributes [26,27], making them suitable for a wide range of experimental studies. Intestinal organoids are developed from pluripotent stem cells, and they have three-dimensional structures. Primary stem cells are able to differentiate into different cell types, but their closed lumen makes drug exposure difficult [28]. Gut-on-a-chip models are a more convenient experimental technique developed on the basis of intestinal organoids [29]. These chips are able to simulate the physiological environment of the intestine, including the fluid and the mechanical forces. The chip is more convenient to cultivate; therefore, it has potential applicability in the development and screening of drugs and bioactive compounds. In this experiment, we tried to construct a model which could simulate the physiology of the human intestine. Finally, we constructed a gut-on-a-chip model with transmembrane resistance, an intestinal physiology, and a three-dimensional cellular morphology similar to the human intestine. The chip can reflect intestinal changes better than a single-layer cell model. In addition, a qPCR analysis showed that the chip we constructed contained several of the most important cell types in the intestine, such as enteroendocrine cells (CHGA) and goblet cells (MUC2). Additionally, the cell types in the chip were similar to the cellular composition of intestinal organoids constructed in other studies [30].

We next continued to induce intestinal cell senescence on the chip. Intestinal senescence is a comprehensive deceleration of both the physiology and function of the intestine, including a decrease in the barrier integrity and an increase in intestinal inflammation [31,32,33]. Ageing of the intestine is always accompanied with a decrease in intestinal cell viability and a gradual increase in intestinal permeability, which lead to a decrease in the transmembrane resistance of the intestinal barrier [34,35]. It has been shown that the cell cycle markers p16 and p21 are linked to cell senescence [36]. Certain studies have used a gut-on-a-chip to model intestinal inflammation and diseases [37]. Some researchers have used gut organoids for ageing-related studies, such as the induction of intestinal ageing via X-ray irradiation or doxorubicin treatment [38]. Based on previous experiments [17], we used H_2_O_2_ to induce senescence of the chip, and attempted simulations to illustrate the mechanisms of intestinal ageing. In our experiment, H_2_O_2_ induced a decrease in the viability of intestinal cells and the TEER of the intestinal barrier, in addition to a disruption of the cell cycle. These results suggest that we have successfully constructed an ageing gut-on-a-chip model.

Intestinal barrier integrity is important for maintaining intestinal homeostasis, but it gradually declines in the process of ageing. Additionally, the content of beneficial commensal microorganisms in the gut decreases with intestinal ageing, leading to dysbiosis and leakage of microbial products. The changes to microbial products promote intestinal inflammation and affect intestinal immune functions [39]. Intestinal leakage caused by the increasing integrity of the intestinal barrier probably triggers inflammatory bowel disease, autoimmune diseases and systemic infectious complications [40]. In this experiment, we observed a decrease in the Claudin-1 content in the H_2_O_2_ model group, which indicates a decrease in the ageing intestinal barrier integrity, although the change was not obvious. We did not find obvious changes in the morphology and structure of the ageing intestinal barrier, which might be due to the faster self-renewal of the intestinal cells [33]. It is said that in the early stage of intestinal ageing, intestinal stem cells can still differentiate and proliferate in the intestinal barrier to some extent [41], so they can compensate for the increased intestinal barrier permeability associated with ageing. As a result, the ageing chip barrier did not exhibit significant damage.

The physiology of intestinal cells also affects the health condition of the intestine. Changes in the levels of cell proliferation reveal the physiological status of the intestine in a sense. A study found that the proliferation rate of crypt cells in the colon of senescent rats increased, and the colon cells showed an over-proliferation [42]. Some studies also found that intestinal stem cells over-proliferate during ageing [43,44]. In this study, the proliferation rate of chip cells in the GOP intervention group was markedly lower compared to the H_2_O_2_ model group, suggesting that the GOP intervention was probably able to slow down the rate of over-proliferation of senescent intestinal cells. A major function of the intestine is secretion, and the mucus is important for intestinal health. Intestinal mucus is also part of the physiological barrier of the intestine. Studies have shown that the proportion of intestinal goblet cells declines markedly [45], as does the content of mucin [24]. Our study found that GOPs were able to increase intestinal mucin expression to some extent, which suggested that GOPs might improve secretion in the ageing intestine.

Ageing is often accompanied by low-grade inflammation in the body. A study has found that an increased intestinal permeability leads to microflora dysbiosis, which ultimately causes intestinal inflammation [4]. However, in our experiment, the content of inflammatory factor IFN-γ in the GOP intervention group exhibited no significant differences compared to the H_2_O_2_ model group, although it seemed a little higher than that of the H_2_O_2_ model group. Therefore, we should explore GOPs’ role in counteracting intestinal inflammation in the future. In addition, mitochondrial dysfunction, which is one of the hallmarks of cellular senescence, also occurs along with the process of ageing [46]. Mitochondrial dysfunction increases intracellular oxidative stress. Furthermore, excessive oxidative stress will damage the normal physiology of intestinal cells. Studies have found that oxidative stress and inflammation are interrelated in many ways [47,48], and it has also been suggested that oxidative stress is associated with inflammatory bowel disease [49]. Studies have also found that the antioxidant capacity of the ageing intestine decreases [50]. In this study, we found that the antioxidant capacity of the ageing chip cells decreased significantly, while the content of antioxidant enzymes in the GOP intervention group increased, which suggests that GOPs can enhance the antioxidant capacity of the intestine. Therefore, we suggest that GOPs might promote intestinal health by improving the antioxidant capacity.

In our research, we constructed a gut-on-a-chip model to better stimulate the physiology of the human gut, and we used H_2_O_2_ treatment to induce cellular senescence so as to explore the effects of interventions on the ageing intestine. This chip model will be used in studies of the ageing intestine, including screening for pharmaceutical and nutritional interventions. Additionally, the chip model enables dynamic observations and mechanistic studies of the ageing intestine. In brief, the model has great potential in applications in the future. Our results revealed that GOP intervention could inhibit the over-proliferation of intestinal cells, increase the secretion of intestinal mucin and improve the antioxidant capacity to maintain intestinal health. A chip model was used in this study; however, there are still some differences between the physiology of the chip and the human intestine. During the experiment, the chip in the ageing model group did not show a large pathological difference compared with the normal control group, and this might be because of the strong renewal ability of the intestinal cells in vitro. Additionally, the short-term intervention might mean that the chip model might not reach the late stage of ageing or that some obvious ageing markers were not detected. We explored GOP intervention effects on an ageing intestine, but we found that GOPs did not have a strong ameliorative effect on the senescent intestine. We suppose that this might be due to the fact that the role of the GOPs is not well represented in the short-term intervention and that we used only one concentration of GOPs. We will continue to improve the experimental methods and select a more suitable molding agent to observe the dynamic changes of the chip so as to better explore the effects of the intervention and the mechanism of action in subsequent studies.

## 5. Conclusions

In our research, we constructed a gut-on-a-chip model which could be important in assessing the role of active substances in food in the future. We found that GOP intervention might maintain intestinal health by regulating the physiology and secretion of intestinal cells, as well as by improving the antioxidant capacity of intestinal cells. As a potential nutrient for health improvement, GOPs are expected to promote intestinal health in the elderly. In addition, gut-on-a-chip models will also be useful in other nutritional studies of the ageing intestine.

## Figures and Tables

**Table 1:** The list of PCR primer base sequences (5′-3′).

Gene	Forward	Reverse
p16	ATGGAGCCTTCGGCTGACT	GTAACTATTCGGTGCGTTGGG
p21	AGGTGGACCTGGAGACTCTCAG	TCCTCTTGGAGAAGATCAGCCG
Ki67	ACGCCTGGTTACTATCAAAAGG	CAGACCCATTTACTTGTGTTGGA
Lgr5	GAGTTACGTCTTGCGGGAAAC	TGGGTACGTGTCTTAGCTGATTA
MUC2	ACTCTCCACACCCAGCATCATC	GTGTCTCCGTATGTGCCGTTGT
P-gp	CCCATCATTGCAATAGCAGG	TGTTCAAACTTCTGCTCCTGA
ZO-1	ACCAGTAAGTCGTCCTGATCC	TCGGCCAAATCTTCTCACTCC
Occludin	TGGGTACGTGTCTTAGCTGATTA	GTCATCCACAGGCGAAGTTAAT
Claudin-1	GTCTTTGACTCCTTGCTGAATCTG	CACCTCATCGTCTTCCAAGCAC

## Data Availability

The data presented in this study are available on request from the corresponding author. The data are not publicly available due to privacy.
